# Boccia as a Rehabilitation Intervention for Adults With Severe Mobility Limitations Due to Neuromuscular and Other Neurological Disorders: Feasibility and Effects on Upper Limb Impairments

**DOI:** 10.3389/fpsyg.2020.00581

**Published:** 2020-03-30

**Authors:** David Suárez-Iglesias, Carlos Ayán Perez, Nuria Mendoza-Laiz, José Gerardo Villa-Vicente

**Affiliations:** ^1^Department of Physical Education and Sport, VALFIS Research Group, Institute of Biomedicine (IBIOMED), University of León, León, Spain; ^2^Well-Move Research Group, Faculty of Education and Sport Science, Department of Special Didactics, University of Vigo, Vigo, Spain; ^3^Department of Sport Science, University of Francisco de Vitoria, Madrid, Spain

**Keywords:** neuromuscular disorders, neurological impairment, severe mobility limitations, upper limb impairments, grip strength, range of motion, Boccia, paralympic

## Abstract

**Purpose:**

Scant research exists regarding the effects of playing Boccia as a rehabilitation strategy for people with severe mobility limitations due to neuromuscular and other neurological disorders. This study is aimed at identifying the feasibility and effects of playing Boccia on the upper limb impairments of people with severe mobility limitations due to neuromuscular and other neurological disorders.

**Materials and Methods:**

Seven people played Boccia three times per week for 20 weeks as part of the rehabilitation process, while other seven kept up with their usual rehabilitation schedule. Attrition, adherence, adverse effects, participation and completion rate were registered to assess feasibility. The effects of the program on grip, pinch strength and upper-limb active range of motion were assessed by means of a dynamometer and a goniometer.

**Results and Conclusions:**

The program was feasible, although no effects were observed after its completion on variables assessed, except for hand flexion and ulnar deviation active range of motion. In a group of people with severe disability due to neuromuscular and other neurological disorders, playing Boccia as part of a multidisciplinary rehabilitation program was shown to be a feasible therapy. However, practicing this game did not lead to significant improvements in upper limb impairments, except for wrist flexion and ulnar deviation active range of motion.

## Introduction

People with neuromuscular and other neurological disorders (NNMDs), including central nervous system disorders such as cerebral palsy (CP) or multiple sclerosis, generally have an unfavorable physical behavior profile characterized by a low level of physical activity, which, in turn, leads to functional deterioration (Slaman et al., 2015; Streber et al., 2016). Practicing adapted sports is considered a beneficial therapeutic approach for this population because it contributes to the development and maintenance of physical function (Shapiro and Malone, 2016). For any adapted sport regimen to be beneficial, it is important to select one that a patient can practice successfully. To this end, there are a number of important person-level characteristics to consider when choosing an adapted sport exercise suitable for people with NNMDs. These factors include degree of disability, economic resources, and motor skill level. In addition, performance of the selected exercise should include sport-specific movements that stimulate and improve conditional aspects that are important not only for sport, but also for daily functioning of the individual.

Boccia is a Paralympic sport originally designed for individuals with CP. The sport, however, is accessible to individuals with other clinical presentations of neurological impairment and other physical impairments. To be eligible to play an individual must experience a disability and use a wheelchair (BISFed, 2018). Boccia’s accessibility presents an interesting option for people with NNMDs for several reasons. Firstly, it is a low-cost, adapted sport modality that is easy to organize, and is accessible to individuals with a very wide range of ability and disability levels (Rimmer, 2012). Given that playing Boccia can be perceived as a recreational form of therapy by those who practice it (Molik et al., 2010), and given that this sport provides a means of control, achievement and identity (Cunningham et al., 2012), adherence to its practice is expected to be high. Therefore, playing Boccia can be regarded as a useful physical rehabilitation strategy for people with severe mobility limitations (CP or related NNMDs involving a wheelchair). Secondly, because Boccia is a precision ball sport, it allows individuals with significant functional limitation to strengthen impaired or weakened muscles and improve their motor skills (Lapresa et al., 2017). Indeed, given that the aim of Boccia is to throw balls closer to a target than an opponent, players that throw the ball with the hand are required to perform a range of motor skills with the upper limb as it involves grabbing, gripping and releasing hand movements (Huang et al., 2014). Therefore, playing Boccia could represent an effective stimulus for improving hand muscular strength and upper extremity range of motion, both of which are conditional aspects that are strongly related to the functional autonomy of people with NNMDs (Yozbatıran et al., 2006; Braendvik et al., 2010).

However, Boccia research has focused mainly on biomechanical, learning and motivational aspects (Barak et al., 2016), while scientific evidence regarding the effects of playing Boccia on upper limb impairments in this population is scarce. This study has a two-fold objective. Firstly, it aims to identify the feasibility of incorporating Boccia as part of a multidisciplinary rehabilitation program for people with severe mobility limitations due to NNMDs. A second goal is to describe changes in hand grip and pinch strength and upper limb range of motion in this population due to the implementation of a Boccia program to their usual rehabilitation program.

## Materials and Methods

### Participants

Participants were recruited from a Spanish State Referral Centre (SRC) for severely disabled people by a Ph.D. student who was carrying out research on the impact of adapted sports in populations with severe disabilities. The study participants were receiving individual level, multidisciplinary, rehabilitation (i.e., psychological and speech therapies and voluntary therapeutic exercise). At the moment of the study, two occupational therapy and two physiotherapy sessions were administered per week, 60 and 30 min each, respectively. While the usual course of rehabilitation treatment was received without interruption, participants were offered the opportunity to practice Boccia as an additional therapy (a leisure time physical activity, LTPA). Those who agreed to participate in a scheduled Boccia program formed the Boccia plus usual rehabilitation program group (BG), and those who did not show interest in participating in regular Boccia sessions formed the usual rehabilitation program group (UG).

All participants with a physical disability affecting all four limbs due to a NNMD or another musculoskeletal disorder, and who used a manual or power wheelchair, were invited to take part in the study. The exclusion criteria were as follows: (a) acute cardiac or respiratory episode in the past 2 months; (b) upper limbs treated with surgery in the past 5 years; (c) engagement in a regular sporting activity (including Boccia) during the previous 4 months; and (d) inability to release the ball. None of the participants exhibited cognitive impairment (Mini-Mental State Examination >26), and all of them were on stable drug therapy for at least three months before and for the period of study participation. A total of 20 participants, with a mean age of 44.2 ± 11.0 years and mean Barthel Index (BI) score of 64.8 ± 29.0, initially volunteered and met the inclusion criteria for the study. Half of them were interested in the Boccia program while the other half were not. The research design was approved by the ethics committees of the University of León and the SRC, and all subjects gave their written informed consent.

### Measurements

#### Baseline Demographic and Clinical Characteristics of Participants

The participants’ age, height, sex, type of disability, functional status (according to the BI), cognitive status (according to the Mini-Mental State Examination) and medication status were obtained from the records of the institution’s medical staff.

#### Feasibility of the Program

A number of variables were collected by the same researcher who administered the program to assess the feasibility of the program. The variables include: recruitment rate (number of participants recruited from those that fulfilled the inclusion criteria), attrition rate (number of drop-outs), completion rate (number of participants who completed each outcome measure), adherence (proportion of participants with participation rates exceeding 80%), participation (total number of session hours completed divided by the total number of possible hours), and safety and tolerability (number of participants who experienced Boccia-related adverse effects).

#### Effects of the Boccia Intervention on Upper-Limb Impairments

##### Maximum grip and pinch strength

Data were collected using Biometrics E-Link H500 Hand Kit that consisted of a G200 Dynamometer with precision load cell and a P200 Pinchmeter for determining grip and pinch strength. Both devices were linked to the E-LINK software (Biometrics Ltd., Gwent, United Kingdom). This system allows accurate measurement on very weak subjects by providing grip and pinch strength measurements to the closest 0.1 increments (kg) (Biometrics Ltd, 2017). The test–retest reliability of grip strength and pinch assessments with the latter has been shown to be good-to-excellent (ICCs 0.83–0.99) in a similar sample of participants as the present study (Hutzler et al., 2013). Calibration accuracy of both G200 Dynamometer and P200 Pinchmeter was verified before and after the test period. Before starting the actual tests, participants were provided with ample opportunity to familiarize themselves with the protocols. An experienced examiner provided consistent encouragement for pre- and post-test, using standardized verbal instructions plus equal feedback for each test and participant (Mathiowetz et al., 1984).

###### Dynamometer test

Participants were tested in their own wheelchairs in accordance with ASHT recommendations (Shechtman and Sindhu, 2015), with shoulder adducted and neutrally rotated, elbow flexed at 90°, and the forearm and wrist in neutral position, while the examiner lightly supported the base of the dynamometer. Participants grasped the dynamometer’s handle that ran parallel to the knuckles with the entire palmar surface of the hand (Mathiowetz et al., 1985). Dynamometer readings present on the Biometrics E-Link’s computer screen were not visible to participants. Standard peak force grip was registered. Participants were instructed to apply as much grip pressure as possible on the dynamometer for 3–5 s. The evaluation system is valid and reliable when used for measuring grip strength with the second handle position (Allen and Barnett, 2011). Thus, the readings of three successive trials were recorded in kilograms using the second handle position of the dynamometer. The three-trial method is recommended in rehabilitation patients with neurologic disorders (Mathiowetz, 1990). A 60-s intertrial rest was established to avoid fatigue. The average result of the three trials was registered for further statistical analysis.

###### Pinchmeter test

After a 10-min break, pinch strength measurements for the key (lateral), three jaw (tripod) and tip to tip positions were performed. The assessment followed the standard procedure and used the recommended three-trial method (Mathiowetz, 1990). For this assessment, participants exert their maximum strength for 3–5 s. The software automatically calculated the average pinch measurement.

##### Range of motion

Active range of motion (AROM) of the upper limb was assessed using an electrogoniometer (Biometrics E-Link R500 Range of Motion Kit), consisting of a small and a large goniometer (Biometrics Ltd, 2018). Specifically, AROM in shoulder (flexion, extension, abduction, medial rotation and lateral rotation), elbow (flexion and extension), forearm (pronation and supination) and wrist (flexion, extension, radial deviation and ulnar deviation) were measured. Test–retest reliability of measuring upper limb ROM in patients with CP has been reported as good (ICCs 0.81–0.94), although it may vary based on the experience of the tester, joint measured, position of the participant and stabilization of the proximal segments (McWhirk and Glanzman, 2006; Mutlu et al., 2007). To control for these factors, standard procedures and positions were followed (Norkin and White, 2016). One experienced examiner took all measurements. For each measurement, the examiner aligned the goniometer and an assistant recorded all data and provided stabilization to participants when required. Three AROM measurements of each motion were taken.

Evaluations took place twice, at baseline (Pre-test) and after a 20-week period (Post-test). All measurements focused on the arm involved in the throwing action and were taken with participants in supine and sitting positions, with a consistent position between tests. The tests were performed under controlled laboratory conditions at the same time of the day and were conducted by the same person who supervised the intervention. The evaluations were performed on two non-consecutive days. Muscular strength measurements were performed on the first day and electrogoniometry was carried out on the second day.

### Procedures

Participants in the BG were asked to attend three Boccia sessions per week for 20 weeks. Fifty-seven sessions were held in a sports room, with two 90-min sessions on Mondays and Wednesdays, and one 60-min session on Thursdays (in total 4 h per week). The sessions included warming-up and cooling-off activities as well as friendly Boccia matches (Table 1). Official Boccia balls were used (weight of 285 ± 12 gr; circumference of 270 mm ± 8 mm). The dimensions and setup of the sports rooms mimic those of a Boccia court (12.5 × 6 m with 2 m of empty space around it), with six rectangular throwing boxes (1 × 2.5 m) in which the players must stay completely within during play. The progression of the Boccia program was based upon participant’s functional ability to play Boccia, performing all activities in their own wheelchairs. To ensure each individual received the required attention and comprehensive instruction tailored to their needs, the following aspects were observed during the first two weeks of the Boccia program by the Boccia coach: a) ability to maintain proper position and balance in the wheelchair (the stability forward, rearward and sideways); static and dynamic postural control before and during the throw, ways of grasping the ball properly (cylindrical, spherical or three-finger grasp), accuracy and coordination in ball-throwing movements (underarm -with or without pendulum movement- and overarm action). From this point on, fundamental movements skills were incorporated into the Boccia sessions as necessary. Moreover, the focus was on the introduction and development of a variety of Boccia-specific skills. This foundation consisted of three phases, that were initiated with simple activities and progressed to more complex activities as the participants advanced their proficiency level in terms of force control, throwing direction and throwing accuracy. The Boccia coach emphasized the improvement of: (a) *force control* (weeks 3–10), where the learning intention was to be able to deliver a ball with the appropriate speed to achieve the intended outcome; (b) *throwing direction* (weeks 11–15), where the learning intention was to apply the principles that underpin delivery of a ball in the right direction toward the intended target, covering different frontal and diagonal throwing lines; and (c) *throwing accuracy* (weeks 16–20), where the learning intention was to refine shot placement and trajectory of shots at targets from different distances, promoting strength and speed of ball-throw accuracy. Participants used different styles of propelling the ball, such as underarm throwing and overarm throwing (Fong et al., 2012), which require wrist, elbow, and shoulder control (Huang et al., 2014). The Boccia program not only promoted skill, but also tactical development. Throughout the 20 weeks, all sessions included games to develop teamwork and the ability to make tactical and strategic decisions. Subjects in both groups were able to throw the ball with their hand, and had never undergone classification evaluation in a sanctioned competition. Participants in the UG group kept up their usual daily activities as part of the specialized multidisciplinary rehabilitation program at the SRC, but did not attend any Boccia sessions.

**TABLE 1:** Activities involved in a typical Boccia group session.

Activity	Duration (min)	Description
**Warm-up**	**10–15**	
Static stretching	3–5	Two sets of isometric holds for 15 s.
Dynamic stretching	3–5	Two sets of repeated upper limb flexion/extension swinging movements for 15 s (i.e., arm circles).
Boccia-specific activities	4–5	Practicing various ways to propel the ball.
**Force control**	**15–20**	
	“Drawing lines”	Each player gets six balls which are thrown to form horizontal lines. This activity is repeated six times, aiming for different distances (short, middle, and long distance) of the boccia court.
**Throwing accuracy**	**15–20**	
	“The Cross Hall”	Three pairs of cones are placed at the 3 m mark. Each pair of cones is separated by a distance equal to the width of two balls. A pair of cones is placed on the right, another in the center and another on the left. The aim is to throw six balls through each pair of cones, making it pass between them without touching.
**Competition simulation**	**15–25**	
		1 vs. 1, 2 vs. 2 or 3 vs. 3 situations.
**Cool-down**	**5–10**	
Static stretching	2–3	Two sets of isometric holds for 15 s.
Relaxation	3–7	Variations of Jacobson’s progressive relaxation exercises.

### Statistical Analysis

Results are presented as mean ± SD. Normality was checked using the Shapiro-Wilk test. All variables were converted to a log-scale, with the corresponding values shown in original units for display purposes. The chi-square test and independent t-test were used to compare clinical characteristics of the two groups. Data from the pre-test-post-test design were compared using two-way repeated measures ANOVA (group × time). The *post hoc* analysis for significant *F*-values was performed with the Bonferroni correction. The assumption of sphericity was evaluated using Mauchly’s test. When this assumption was not met, the level of significance was adjusted by means of the Greenhouse-Geisser epsilon. Additionally, an independent t-test was used to compare the measures of maximum grip and pinch strength and range of motion between the two groups at pre- and post-test. Partial eta squared (η_*p*_^2^) values were calculated as indicators of effect size, and values of 0.01, 0.06, and 0.14 were considered small, moderate, and large effect sizes, respectively (Cohen, 1988). Magnitude-based inferences were used for analysis of clinical significance using a published spreadsheet (Hopkins, 2006). The threshold for a change to be considered clinically important (smallest worthwhile change, SWC) was set as 0.2 × observed between participant SD, based on Cohen’s d effect size principle (Batterham and Hopkins, 2006). The magnitude of difference was expressed as the standardized mean difference. The probability that the magnitude of change was greater than the SWC was rated as: <0.5% almost certainly not; 0.6–5% very unlikely; 6–25% unlikely; 26–75% possibly; 76–95% likely; 96–99.5% very likely; >99.5% most likely. For a clinical inference, the effect was indicated as “unclear” if its chance of benefit is promising but its risk of harm is unacceptable; the effect was otherwise characterized by a statement about the chance that it is “trivial”, “beneficial” or “harmful” (% chances: beneficial/trivial/harmful%) (Hopkins, 2006). The statistical significance was set at *p* < 0.05. All analyses were performed using SPSS v. 22.0 for Windows.

## Results

Out of the 20 people who initially met the inclusion criteria, two dropped out. One participant included in the BG moved away from the SRC, and the other, included in the NG, dropped out due to family reasons. Therefore, the recruitment rate for the BG was 90% (9/10). Four subjects (two from the BC and two from the UG) were omitted from further analysis due to missing data, as they were unable to perform the baseline tests as requested. Thus, a completion rate of 77.7% (7/9) was obtained for the BG. Two participants attended less than 80% of the scheduled sessions (71.9% and 78.9%, respectively). Therefore, adherence to the program in the BG stood at 71.4% (5/7). All scheduled Boccia practice hours were completed, and no injuries or adverse effects were registered. The duration of the sessions did not affect the observed adherence values.

The final study sample was made up of 14 people (mean age of 44.1 ± 10.8 years; mean BI score of 64.3 ± 16.2; 64% women) (Table 2). The BG (mean age of 44.1 ± 12.6 years; mean BI score of 72.9 ± 14.7) included five women (CP, *n* = 2; Friedreich’s ataxia, *n* = 1; poliomyelitis, *n* = 1; Charcot-Marie-Tooth disease, *n* = 1) and two men (ataxia, *n* = 1; Steiner’ myotonic dystrophy, *n* = 1). The UG group (mean age of 44.1 ± 9.8 years; mean BI score of 55.7 ± 13.4) included four women (CP, *n* = 3; traumatic brain injury, *n* = 1) and three men (CP, *n* = 2; neurofibromatosis, *n* = 1). All participants presented an impairment that belongs to at least one of the six Boccia eligible impairment types identified in the Boccia Classification Rulebook (BISFed, 2018).

**TABLE 2:** Baseline participant characteristics and adherence to Boccia program.

Participant	Age	Gender	Diagnosis	Time since diagnosis	Barthel Index	Wheelchair type	Presence (no of sessions)	Absence (no of sessions)	Reasons for absence	Adherence (%)
***Boccia plus usual rehabilitation program***										
1	42	Female	FA	17	90	Manual	57	0		100.0
2	54	Female	CP	54	50	Power	55	2	(2) visit family	96.5
3	21	Female	CP	21	85	Power	55	2	(3)	96.8
4	56	Female	Polio	50	65	Power	53	4	(4) illness: flu	93.0
5	35	Male	FA	11	80	Manual	50	7	(6) health issues	87.7
6	48	Female	CMT	14	60	Power	45	12	(10) health issues	78.9
7	53	Male	DM1	9	80	Power	41	16	(12) health issues	71.9
***Usual rehabilitation program***										
8	62	Male	CP	62	60	Power				
9	33	Female	CP	33	65	Power				
10	52	Male	NF1	52	60	Manual				
11	40	Male	CP	40	75	Manual				
12	39	Female	CP	39	50	Power				
13	44	Female	TBI	4	35	Power				
14	39	Female	CP	39	45	Power				

**Abbreviation*: FA, Friedreich’s ataxia; CP, cerebral palsy; Polio, poliomyelitis; CMT, Charcot-Marie-Tooth disease; DM1, myotonic dystrophy type 1; NF1, neurofibromatosis type I; TBI, traumatic brain injury.*

The groups were comparable at baseline on the relevant baseline characteristics (except for BI score, *p* = 0.041), and on all tested variables except for pinch strength values: key position (*p* = 0.035), three jaw position (*p* = 0.025), and tip to tip position (*p* = 0.046). The intragroup analysis indicated that neither group experienced significant changes in the variables assessed after the intervention, except for wrist flexion and ulnar deviation, which significantly improved in the BG (Table 3).

**TABLE 3:** Between-group analysis.

Variables		Boccia plus usual rehabilitation program								Usual rehabilitation program								
		1	2	3	4	5	6	7	M ± SD	8	9	10	11	12	13	14	M ± SD	*p*
**Grip strength (N)**	Pre-test	196.1	84.3	88.3	117.7	313.8	147.1	84.3	157.986.9	176.5	137.3	186.3	156.9	186.3	156.9	186.3	158.935.1	0.221
	Post-test	211.8	81.4	81.4	109.8	360.9	166.7	80.4	168.7107.2	188.3	166.7	147.1	166.7	127.5	225.6	221.6	165.548.3	0.272
	Change	15.7	–2.9	–6.9	–7.8	47.1	19.6	–3.9	8.720.2	11.8	29.4	–39.2	9.8	–58.8	68.6	35.3	8.143.9	0.976
**Pinch strength (N)**																		
*Key*	Pre-test	31.4	30.4	23.5	14.7	16.7	40.2	19.6	26.19.7	22.6	32.4	58.8	34.3	30.4	41.2	52.0	25.35.2	**0**.**035**
	Post-test	26.5	22.6	16.7	25.5	30.4	30.4	23.5	36.413.6	46.1	46.1	51.0	61.8	19.6	38.2	30.4	39.614.4	**0**.**014**
	Change	–4.9	–7.8	–6.9	10.8	13.7	–9.8	3.9	−0.19.6	23.5	13.7	–7.8	27.5	–10.8	–2.9	–21.6	3.118.6	0.690
*Three jaw*	Pre-test	11.8	30.4	17.7	15.7	14.7	47.1	13.7	22.913.5	16.7	31.4	58.8	28.4	36.3	48.1	59.8	36.617.6	**0**.**025**
	Post-test	12.7	33.3	16.7	24.5	25.5	40.2	11.8	25.510.2	42.2	34.3	56.9	46.1	22.6	43.2	38.2	36.914.1	**0**.**014**
	Change	1.0	2.9	–1.0	8.8	10.8	–6.9	–2.0	1.96.2	25.5	2.9	–2.0	17.7	–13.7	–4.9	–21.6	0.619.6	0.861
*Tip to tip*	Pre-test	10.8	28.4	14.7	13.7	28.4	37.3	13.7	22.210.6	28.4	17.7	41.2	26.5	38.2	29.4	41.2	29.510.3	**0**.**046**
	Post-test	8.8	25.5	18.6	14.7	25.5	37.3	7.8	21.710.0	30.4	26.5	35.3	39.2	11.8	35.3	38.2	28.112.0	0.078
	Change	–2.0	–2.9	3.9	1.0	–2.9	0.0	–5.9	−1.33.2	2.0	8.8	–5.9	12.7	–26.5	5.9	–2.9	−0.813.0	0.936
**Shoulder AROM (°)**																		
Flexion	Pre-test	138	120	119	152	60	69	144	114.636.3	65	144	163	132	113	142	63	88.957.1	0.928
	Post-test	139	140	143	155	141	138	37	127.640.3	74	145	135	160	86	136	69	117.439.4	0.763
	Change	1	20	24	3	81	69	–107	13.061.3	9	1	–28	28	–27	–6	6	−2.420.0	0.539
Extension	Pre-test	14	9	23	13	30	14	28	18.78.2	–12	48	23	10	5	11	16	14.418.4	0.646
	Post-test	17	18	29	40	30	30	17	25.98.8	8	16	–2	26	15	–15	9	8.113.3	**0**.**029**
	Change	3	9	6	27	0	16	–11	7.112.1	20	–32	–25	16	10	–26	–7	−6.321.8	0.180
Abduction	Pre-test	75	50	47	80	79	37	74	63.117.8	45	84	77	76	68	68	39	65.316.9	0.816
	Post-test	73	62	76	92	110	65	39	73.922.6	91	18	53	84	80	32	33	55.929.3	0.174
	Change	–2	12	29	12	31	28	–35	10.723.5	46	–66	–24	8	12	–36	–6	−9.436.4	0.242
Medial rotation	Pre-test	28	18	3	19	65	50	27	30.020.9	1	50	17	31	59	30	18	29.420.0	0.811
	Post-test	53	40	20	18	70	56	27	40.619.9	15	25	29	27	36	67	32	33.016.4	0.504
	Change	25	22	17	–1	5	6	0	9.08.8	14	–25	12	–4	–23	37	14	0.722.0	0.374
Lateral rotation	Pre-test	127	63	41	122	77	78	20	75.439.3	61	65	83	62	75	73	76	70.78.2	0.749
	Post-test	99	54	86	117	104	73	90	89.020.8	70	97	76	131	102	62	29	81.032.7	0.469
	Change	–28	–9	45	–5	27	–5	70	13.634.8	9	32	–7	69	27	–11	–47	10.337.0	0.867
**Elbow AROM (°)**																		
*Flexion*	Pre-test	160	143	108	118	124	122	53	118.333.6	67	109	138	88	127	96	114	105.624.1	0.587
	Post-test	115	139	120	115	137	93	89	115.419.3	151	78	112	148	95	89	107	111.428.3	0.672
	Change	–45	–4	12	–3	13	–29	36	−2.927.2	84	–31	–26	60	–32	–7	–7	5.946.9	0.678
*Extension*	Pre-test	–44	–33	–58	–14	1	–21	–25	−27.719.5	4	–4	–3	–25	–23	–5	–29	−12.113.1	0.105
	Post-test	–43	–10	–29	–37	–41	–41	–34	−33.611.5	–14	2	–21	–10	–14	–17	–42	−16.613.3	**0**.**025**
	Change	1	23	29	–23	–42	–20	–9	−5.925.5	–18	6	–18	15	9	–12	–13	−4.413.9	0.899
**Forearm AROM (°)**																		
*Pronation*	Pre-test	75	75	55	75	75	75	54	69.110.0	77	64	72	62	66	75	56	67.47.6	0.782
	Post-test	75	75	70	71	75	75	34	67.915.1	75	75	75	64	65	75	29	65.416.8	0.784
	Change	0	0	15	–4	0	0	–20	−1.310.2	–2	11	3	2	–1	0	–27	−2.011.8	0.906
*Supination*	Pre-test	60	72	70	79	71	77	77	72.36.4	72	70	75	70	76	79	65	72.44.6	0.931
	Post-test	62	75	76	75	75	75	75	73.35.0	70	75	73	75	73	75	66	72.43.4	0.754
	Change	2	3	6	–4	4	–2	–2	1.03.7	–2	5	–2	5	–3	–4	1	0.03.7	0.624
**Wrist AROM (°)**																		
*Flexion*	Pre-test	42	51	22	44	73	32	33	42.416.5	64	34	58	62	57	56	45	53.710.6	0.122
	Post-test	52	48	67	50	74	46	73	58.612.3	70	30	62	73	57	55	63	58.614.2	0.918
	Change	10	–3	45	6	1	14	40	16.118.9	6	–4	4	11	0	–1	18	4.97.6	0.169
*Extension*	Pre-test	38	18	24	53	76	42	13	37.722.0	48	85	76	85	38	28	83	63.324.5	0.064
	Post-test	47	15	31	74	70	20	66	46.124.6	47	63	20	44	56	77	63	52.918.2	0.465
	Change	9	–3	7	21	–6	–22	53	−3.724.0	–1	–22	–56	–41	18	49	–20	0.924.7	0.731
*Radial deviation*	Pre-test	30	24	16	30	30	30	9	24.18.5	28	30	30	18	9	29	38	26.09.5	0.794
	Post-test	13	24	18	18	30	31	44	25.410.5	30	35	20	12	17	19	37	24.39.7	0.841
	Change	–17	0	2	–12	0	1	35	1.316.6	2	5	–10	–6	8	–10	–1	−1.77.2	0.668
*Ulnar deviation*	Pre-test	11	18	3	3	16	2	31	12.010.6	31	6	27	17	28	36	23	24.09.9	0.052
	Post-test	35	31	36	47	50	31	32	37.47.8	24	17	32	22	10	45	24	24.911.2	**0**.**029**
	Change	24	13	33	44	34	29	1	25.414.4	–7	11	5	5	–18	9	1	0.910.2	**0**.**003**

*AROM: Active Range of Motion. In bold, significant changes where, *p* < 0.05.*

The clinical inference analysis showed that the observed changes were mostly unclear or trivial, except for shoulder medial rotation (90% CI of difference 11.0 ± 12.0; *p* = 0.13; η_*p*_^2^ = 0.11; *F* = 2.51; 83/15/2%), wrist flexion (90% CI of difference 16.0 ± 11.0; *p* = 0.01; η_*p*_^2^ = 0.27; *F* = 7.44; 97/3/0%) and ulnar deviation (90% CI of difference 25.0 ± 11.0; *p* < 0.001; η_*p*_^2^ = 0.43; *F* = 14.96; 100/0/0%), which were classified as likely to be beneficial, very likely to be beneficial and most likely to be beneficial, respectively.

## Discussion

Knowledge of the feasibility and effects of exercise programs in NNMDs is required in order to provide evidence-based recommendations. In this study, Boccia was shown to be a feasible physical therapy for people with severe disability due to NNMDs. Indeed, although a few drop outs were registered, none of them were related to the proposed activities. Moreover, no adverse events were registered and adherence to the intervention was acceptable, and similar to those observed in other exercise interventions performed on this population (Kierkegaard et al., 2011). This is a finding worth of mentioning, since adherence and drop-outs are possible threats to the validity and outcome of any intervention study, but especially those carried out in people with NNMDs (Aldehag et al., 2013).

The feasibility and adherence observed in the present study can be explained on the bases of institutional, interpersonal and intrapersonal factors. Concerning institutional factors, research suggests that a health professional’s skill and knowledge in terms of exercise guidance, building/facility accessibility, lack of transportation and low and limited financial resources, are aspects that strongly affect participation in people with physical disabilities (Martin Ginis et al., 2016). In the present study, and in accordance with suggestions to increase participation in this kind of interventions (Shirazipour et al., 2018), an exercise specialist with strong physical activity and disability-specific knowledge monitored the sessions. Moreover, the building and the facilities in which the intervention took place were completely accessible to people with mobility limitations. It is also important to note that transportation was provided for those participants who needed this service, and the Boccia activity was free of charge. Thus, transportation and cost barriers were properly addressed, so adherence in the program could have consequently improved due to this strategy. For instance, in people with severe multiple sclerosis who participated in an exercise program free of charge and in which transportation was arranged and paid for, a high adherence rate was observed (van der Linden et al., 2014). Regarding interpersonal factors (i.e., self-perceived support and attitudes) the fact that the Boccia group was exclusively comprised of participants with similar functional limitations could have improved adherence to the program, since the opportunity for participants to meet and exercise with others with similar disability levels has been acknowledged as an important point to consider in this regard (Kierkegaard et al., 2011). Indeed, it has been suggested that in patients with NNMDs, social and behavioral aspects improve markedly when training programs are held in groups where people with the same condition (i.e., sclerosis multiple) share experiences and make friends (Sánchez-Lastra et al., 2019), as in the case of this study. Fatigue, lack of motivation and apathy are intrapersonal factors that influence exercise adherence for people with NNMDs (Aldehag et al., 2013). Other barriers to exercise for this population include exercise complexity (Phillips et al., 2009) and activity related embarrassment (Martin Ginis et al., 2016). Therefore, because of the fact that Boccia sessions included friendly matches, easy to perform tasks, and were conducted in a recreational, relaxed atmosphere; it could have had a positive impact on the adherence to the program. The lack of adverse effects observed could also be due to this reason, as few harmful effects related to Boccia have been observed in those who play at a competitive level (Fong et al., 2012). Finally, it should not be overlooked that participants in the Boccia group wanted to practice this sport, thus, they were probably motivated to undertake the intervention and inclined to persevere. Taken together, these findings suggest that the degree of disability showed by the participants in this study may not have caused significant barriers to their desire and ability to practice Boccia as as rehabilitation strategy.

Nevertheless, we acknowledge the effects of the Boccia program on the participants’ hand muscular strength and upper limb range of motion are anecdotal at best. This is because this was not a randomized controlled research and the groups were not fully comparable at baseline. Both facts constitute a potential source of bias, therefore we are cautious about making inferences from this study. Our findings suggest that playing Boccia did not improve hand grip muscular strength. We believe the lack of effect could be due to the nature of the performed activity, as research suggests, improvements in muscular hand strength have been observed in people with NNMDs who carried out specific and individualized strengthening exercise programs have been observed (Anziska and Sternberg, 2013; Hutzler et al., 2013). Other researchers have reported positive effects of playing games (Wii training) on the hand grip muscular strength of people with upper limb impairments. It should be noted that the proposed intervention also included strengthening exercises and activities aimed at improving hand skills (El-Shamy and El-Banna, 2018). In the light of all this, it seems that in order to achieve improvements in hand muscular strength, people with NNMDs should take part in muscular training programs specifically designed for this purpose.

In people with NNMDs, upper limb impairment results in reduced manual dexterity, which interferes with the execution of daily life activities. As tripod pinch strength and thumb opposition are major determinants of manual dexterity, it seems important to develop rehabilitation strategies aimed at increasing their functionality (Videler et al., 2010). However, few investigations have been carried out in this regard, specifically in people with severe disability. In the present study, playing Boccia did not lead to changes in pinch strength, implying that this game does not have a positive impact on this variable in people with NMD. Improvements on the pinch strength level of this population have been found after the performance of exercise programs specifically designed for this purpose (Hutzler et al., 2013; Regardt et al., 2014; El-Shamy and El-Banna, 2018). Therefore, one can assume that when it comes to improving pinch strength for people with NNMDs, performing targeted exercise of specific muscles may be a more effective rehabilitation approach. However, Aldehag et al. (2013) did not find any significant changes in the pinch grip force of a group of people with myotonic dystrophy, even after completing a 12-weeks hand training program specifically designed for people with this disability. Together, these results suggest that although pinch strength can be improved by means of specific muscular training programs, not all people with NNMDs will benefit from them. Thus, the existing heterogeneity in NNMDs should be taken into account when designing this type of rehabilitation strategy.

Rehabilitation strategies for NNMDs should include therapies aimed at improving AROM, however, scientific evidence is scarce (Johnson et al., 2012). The findings of this study provide preliminary evidence regarding the effects of playing Boccia on AROM for people with severe upper limb functional limitation. After the intervention, some positive changes were observed in hand wrist flexion and ulnar deviation. The MBI method showed that both changes were considered very likely or most likely to be beneficial. Interestingly, a strong association between ulnar deviation and wrist flexion range of motion has been observed (Li et al., 2005), suggesting that changes in one movement influences the range of motion in the other. Given that wrist flexion and ulnar deviation are related to spasticity (de Bruin et al., 2014), it could be hypothesized that playing Boccia could be a useful strategy to ameliorate this symptom. In this regard, further research is needed.

Patients with NNMDs have reported that they would like to see more research into movement and physical training (Jerath et al., 2017). Although no major improvements or detrimental effects in the upper limb impairments tested in this study were observed (hand strength, shoulder, elbow and hand range of motion), the findings add to the scientific body of knowledge. Moreover, very limited information has been reported to date on the impact of a training program on the upper limb impairments in people with severe disability due to NNMDs. Similarly, limited research has been carried out on the effects of playing Boccia as a physical therapy rehabilitation strategy. Therefore, the main strength of this study lies in its originality, on which future randomized trials aimed at increasing the existing scientific evidence in the field of physical rehabilitation for people with severe upper limb impairments can be based. However, we acknowledge that a small number of participants took part in this non-randomized, and they were not randomly distributed between groups. Both factors increase the risk of bias and the probability of making a type II error. Therefore, future randomized trials with larger samples are needed to confirm the results showed here. It should also be noted that the evaluation was not blind. In addition, although all participants showed similar upper limb impairments, the origin of their disability was equally varied, implying the existence of certain heterogeneity in the BG and UG groups. In this regard, we evaluated the effects of the Boccia program on the participants’ muscular strength and range of motion, however, we did not identify the impact that the program had on their functional independence level. Finally, we did not apply a progression in training load and variation in stimulus regarding size and weight of the Boccia balls. These limitations should be taken into account when interpreting the findings of this study.

## Conclusion

In a group of people with severe disability due to NNMDs, playing Boccia as part of a multidisciplinary rehabilitation program was shown to be a feasible therapy. However, practicing this game did not lead to significant improvements in upper limb impairments, except for wrist flexion and ulnar deviation active range of motion. Future randomized controlled trials with a larger sample size are needed to confirm these findings.

## Data Availability Statement

All datasets generated for this study are included in the article/supplementary material.

## Ethics Statement

The studies involving human participants were reviewed and approved by Comité de Ética de la Universidad de León. The patients/participants provided their written informed consent to participate in this study.

## Author Contributions

NM-L and JV-V contributed to the conception and design of the study. All authors were taken part in the acquisition, analysis, and interpretation of data, contributed to drafting the article and critically revised the article for important intellectual content, and DS-I and CA approved the last version to be published.

## Conflict of Interest

The authors declare that the research was conducted in the absence of any commercial or financial relationships that could be construed as a potential conflict of interest.

## References

[B1] Aldehag A. Jonsson H. Lindblad J. Kottorp A. Ansved T. Kierkegaard M. (2013). Effects of hand-training in persons with myotonic dystrophy type 1 – a randomised controlled cross-over pilot study. *Disabil. Rehabil.* 35 1798–1807. 10.3109/09638288.2012.754952 23480644

[B2] Allen D. Barnett F. (2011). Reliability and validity of an electronic dynamometer for measuring grip strength. *Int. J. Ther. Rehabil.* 18 258–264. 10.12968/ijtr.2011.18.5.258

[B3] Anziska Y. Sternberg A. (2013). Exercise in neuromuscular disease. *Muscle Nerve* 48 3–20. 10.1002/mus.23771 23695822

[B4] Barak S. Mendoza-Laiz N. Fuentes M. T. G. Rubiera M. Huyzler Y. (2016). Psychosocial effects of competitive boccia program in persons with severe chronic disability. *J. Rehabil. Res. Dev.* 53 973–988. 10.1682/JRRD.2015.08.0156 28475199

[B5] Batterham A. M. Hopkins W. G. (2006). Making meaningful inferences about magnitudes. *Int. J. Sports Physiol. Perform.* 1 50–57. 10.1123/ijspp.1.1.50 19114737

[B6] Biometrics Ltd (2017). *Hand Kit (Grip & Pinch Dynamometer).* Available online at: http://www.biometricsltd.com/h500.htm (accessed November 26, 2019).

[B7] Biometrics Ltd (2018). *Range of Motion Kit (Goniometers).* Available online at: http://www.biometricsltd.com/range-of-motion-kit.htm (accessed November 26, 2019).

[B8] BISFed (2018). *Boccia Classification Rules*, 4th Edn Available online at: http://www.bisfed.com/wp-content/uploads/2018/12/Boccia-Classification-Rules-4th-Edition-October-2018.pdf (accessed November 26, 2019).

[B9] Braendvik S. M. Elvrum A.-K. G. Vereijken B. Roeleveld K. (2010). Relationship between neuromuscular body functions and upper extremity activity in children with cerebral palsy. *Dev. Med. Child Neurol.* 52 e29–e34. 10.1111/j.1469-8749.2009.03490.x 19811515

[B10] Cohen J. (1988). *Statistical Power Analysis for the Behavioral Sciences*, 2nd Edn Hillsdales, NJ: Lawrence Erlbaum Associates.

[B11] Cunningham C. Wensley R. Blacker D. Bache J. Stonier C. (2012). Occupational therapy to facilitate physical activity and enhance quality of life for individuals with complex neurodisability. *Br. J. Occup. Ther.* 75 106–110. 10.4276/030802212X13286281651234

[B12] de Bruin M. van de Giessen M. Vroemen J. C. Veeger H. E. J. Maas M. Strackee S. D. (2014). Geometrical adaptation in ulna and radius of cerebral palsy patients: measures and consequences. *Clin. Biomech.* 29 451–457. 10.1016/j.clinbiomech.2014.01.003 24485089

[B13] El-Shamy S. M. El-Banna M. F. (2018). Effect of Wii training on hand function in children with hemiplegic cerebral palsy. *Physiother. Theory Pract.* 36 38–44. 10.1080/09593985.2018.1479810 29792556

[B14] Fong D. T.-P. Yam K.-Y. Chu V. W.-S. Cheung R. T.-H. Chan K.-M. (2012). Upper limb muscle fatigue during prolonged Boccia games with underarm throwing technique. *Sports Biomech.* 11 441–451. 10.1080/14763141.2012.699977 23259234

[B15] Hopkins W. G. (2006). Spreadsheets for analysis of controlled trials, with adjustment for a subject characteristic. *Sportscience* 10 46–50.

[B16] Huang P.-C. Pan P.-J. Ou Y.-C. Yu Y.-C. Tsai Y.-S. (2014). Motion analysis of throwing boccia balls in children with cerebral palsy. *Res. Dev. Disabil.* 35 393–399. 10.1016/j.ridd.2013.11.017 24334228

[B17] Hutzler Y. Lamela B. Mendoza N. Díez I. Barak S. (2013). The effects of an exercise training program on hand and wrist strength, and function, and activities of daily living, in adults with severe cerebral palsy. *Res. Dev. Disabil.* 34 4343–4354. 10.1016/j.ridd.2013.09.015 24145046

[B18] Jerath N. U. Simoens K. Mann D. Kollasch S. Grosland N. Malik K. A. (2017). Survey of the functional priorities in patients with disability due to neuromuscular disorders. *Disabil. Rehabil. Assist. Technol.* 14 133–137. 10.1080/17483107.2017.1413143 29216771

[B19] Johnson L. B. Florence J. M. Abresch R. T. (2012). Physical therapy evaluation and management in neuromuscular diseases. *Phys. Med. Rehabil. Clin. N. Am.* 23 633–651. 10.1016/j.pmr.2012.06.005 22938879

[B20] Kierkegaard M. Harms-Ringdahl K. Edström L. Widén Holmqvist L. Tollbäck A. (2011). Feasibility and effects of a physical exercise programme in adults with myotonic dystrophy type 1: a randomized controlled pilot study. *J. Rehabil. Med.* 43 695–702. 10.2340/16501977-0833 21670942

[B21] Lapresa D. Santesteban G. Arana J. Anguera M. T. Aragón S. (2017). Observation system for analyzing individual boccia BC3. *J. Dev. Phys. Disabil.* 29 721–734. 10.1007/s10882-017-9552-2

[B22] Li Z.-M. Kuxhaus L. Fisk J. A. Christophel T. H. (2005). Coupling between wrist flexion–extension and radial–ulnar deviation. *Clin. Biomech.* 20 177–183. 10.1016/j.clinbiomech.2004.10.002 15621323

[B23] Martin Ginis K. A. Ma J. K. Latimer-Cheung A. E. Rimmer J. H. (2016). A systematic review of review articles addressing factors related to physical activity participation among children and adults with physical disabilities. *Health Psychol. Rev.* 10 478–494. 10.1080/17437199.2016.1198240 27265062

[B24] Mathiowetz V. (1990). Effects of three trials on grip and pinch strength measurements. *J. Hand Ther.* 3 195–198. 10.1016/S0894-1130(12)80377-2

[B25] Mathiowetz V. Kashman N. Volland G. Weber K. Dowe M. Rogers S. (1985). Grip and pinch strength: normative data for adults. *Arch. Phys. Med. Rehabil.* 66 69–74. 3970660

[B26] Mathiowetz V. Weber K. Volland G. Kashman N. (1984). Reliability and validity of grip and pinch strength evaluations. *J. Hand Surg. Am.* 9 222–226. 10.1016/S0363-5023(84)80146-X 6715829

[B27] McWhirk L. B. Glanzman A. M. (2006). Within-session inter-rater realiability of goniometric measures in patients with spastic cerebral palsy. *Pediatr. Phys. Ther.* 18 262–265. 10.1097/01.pep.0000234960.88761.97 17108799

[B28] Molik B. Zubala T. Słyk K. Bigas G. Gryglewicz A. Kucharczyk B. (2010). Motivation of the disabled to participate in chosen Paralympics events (wheelchair basketball, wheelchair rugby, and boccia). *Fizjoterapia* 18 42–51. 10.2478/v10109-010-0044-5

[B29] Mutlu A. Livanelioglu A. Gunel M. K. (2007). Reliability of goniometric measurements in children with spastic cerebral palsy. *Med. Sci. Monit.* 13 CR323–CR329. 17599027

[B30] Norkin C. C. White D. J. (2016). *Measurement of Joint Motion: A Guide to Goniometry*, 5th Edn Philadelphia, PA: F.A. Davis Company.

[B31] Phillips M. Flemming N. Tsintzas K. (2009). An exploratory study of physical activity and perceived barriers to exercise in ambulant people with neuromuscular disease compared with unaffected controls. *Clin. Rehabil.* 23 746–755. 10.1177/0269215509334838 19506002

[B32] Regardt M. Schult M.-L. Axelsson Y. Aldehag A. Alexanderson H. Lundberg I. E. (2014). Hand exercise intervention in patients with polymyositis and dermatomyositis: a pilot study. *Musculoskeletal Care* 12 160–172. 10.1002/msc.1069 24623733

[B33] Rimmer P. (2012). Boccia–“follow your dream and you can do anything”. *Palaestra* 26 31–34.

[B34] Sánchez-Lastra M. A. Martínez-Aldao D. Molina A. J. Ayán C. (2019). Pilates for people with multiple sclerosis: a systematic review and meta-analysis. *Mult. Scler. Relat. Disord.* 28 199–212. 10.1016/j.msard.2019.01.006 30623859

[B35] Shapiro D. R. Malone L. A. (2016). Quality of life and psychological affect related to sport participation in children and youth athletes with physical disabilities: a parent and athlete perspective. *Disabil. Health J.* 9 385–391. 10.1016/j.dhjo.2015.11.007 26747413

[B36] Shechtman O. Sindhu B. (2015). “Grip strength dynamometry,” in *Clinical Assessment Recommendations*, 3rd Edn, ed. McDermid J. (Mount Laurel, NJ: American Society of Hand Therapists).

[B37] Shirazipour C. H. Evans M. B. Leo J. Lithopoulos A. Martin Ginis K. A. Latimer-Cheung A. E. (2018). Program conditions that foster quality physical activity participation experiences for people with a physical disability: a systematic review. *Disabil. Rehabil.* 42 147–155. 10.1080/09638288.2018.1494215 30324815

[B38] Slaman J. Roebroeck M. Dallmijer A. Twisk J. Stam H. van den Berg-Emons R. (2015). Can a lifestyle intervention programme improve physical behaviour among adolescents and young adults with spastic cerebral palsy? A randomized controlled trial. *Dev. Med. Child Neurol.* 57 159–166. 10.1111/dmcn.12602 25303096

[B39] Streber R. Peters S. Pfeifer K. (2016). Systematic review of correlates and determinants of physical activity in persons with multiple sclerosis. *Arch. Phys. Med. Rehabil.* 97 633–645.e29. 10.1016/j.apmr.2015.11.020 26751247

[B40] van der Linden M. L. Bulley C. Geneen L. J. Hooper J. E. Cowan P. Mercer T. H. (2014). Pilates for people with multiple sclerosis who use a wheelchair: feasibility, efficacy and participant experiences. *Disabil. Rehabil.* 36 932–939. 10.3109/09638288.2013.824035 23957639

[B41] Videler A. J. Beelen A. van Schaik I. N. Verhamme C. van den Berg L. H. de Visser M. (2010). Tripod pinch strength and thumb opposition are the major determinants of manual dexterity in Charcot-Marie-Tooth disease type 1A. *J. Neurol. Neurosurg. Psychiatry* 81 828–833. 10.1136/jnnp.2009.187302 20562406

[B42] Yozbatıran N. Baskurt F. Baskurt Z. Ozakbas S. Idiman E. (2006). Motor assessment of upper extremity function and its relation with fatigue, cognitive function and quality of life in multiple sclerosis patients. *J. Neurol. Sci.* 246 117–122. 10.1016/j.jns.2006.02.018 16678208

